# Autotransplantations Without Cryopreservation

**DOI:** 10.1200/JGO.18.00021

**Published:** 2018-02-28

**Authors:** Guillermo J. Ruiz-Argüelles, Robert Peter Gale

**Affiliations:** **Guillermo J. Ruiz-Argüelles**, Clínica Ruiz, Puebla, México; and **Robert Peter Galed**, Imperial College London, London, United Kingdom.

## TO THE EDITOR:

The article by Majolino et al^1^ describes an autotransplantation program that has been started in Iraq and that could be used as an example by which to start similar programs in other developing countries. Developing a facility to freeze and store hematopoietic cells—blood or bone marrow—is complex and expensive and requires substantial expertise. We studied whether hematopoietic cells could be stored at 4°C in a multicenter analysis of 359 participants from México, Colombia, and Argentina, with plasma cell myeloma (n = 216) and lymphomas (n = 143).^2^ Blood cells were mobilized with filgrastim and collected after a median of two aphereses. Apheresis products were stored at 4°C for a median of 3 d. The median number of mononuclear cells was 31 × 10E+6/kg, median number of CD34-positive cells was 3.6 × 10E+6/kg, and median viability after collection was 90% after storage. All except one evaluable participant recovered bone marrow function, and there was no late bone marrow failure. Median interval to neutrophils > 0.5 × E+9/L was 13 d, and to platelets > 20 × 10E+9/L was 16 d. There was no correlation between the number of storage days at 4°C and viability after storage, nor rates of recovery of neutrophils or platelets. Others have reported similar data.^3^ Blood cells that were collected for autotransplantation can be stored at 4°C for 6 d, and storing hematopoietic cells at 4°C could expand autotransplantations to centers in developing countries where more complex technical skills and equipment are lacking and/or cost may be an issue. It could also reduce complexity and cost at centers that presently freeze cells for an autotransplantation.
